# Exploring the CD3/CD56/TNF-α/Caspase3 pathway in pyrethroid-induced immune dysregulation: curcumin-loaded chitosan nanoparticle intervention

**DOI:** 10.3389/fphar.2025.1505432

**Published:** 2025-02-06

**Authors:** Nawal Alsubaie, Yasmina M. Abd-Elhakim, Amany Abdel-Rahman Mohamed, Tarek Khamis, Mohamed M. M. Metwally, Nawal Helmi, Afnan M. Alnajeebi, Badriyah S. Alotaibi, Amirah Albaqami, Wedad Mawkili, Mai A. Samak, Samar A. Eissa

**Affiliations:** ^1^ Department of Pharmacy Practice, College of Pharmacy, Princess Nourah bint Abdulrahman University, Riyadh, Saudi Arabia; ^2^ Department of Forensic Medicine and Toxicology, Faculty of Veterinary Medicine, Zagazig University, Zagazig, Egypt; ^3^ Department of Pharmacology, Faculty of Veterinary Medicine, Zagazig University, Zagazig, Egypt; ^4^ Laboratory of Biotechnology, Faculty of Veterinary Medicine, Zagazig University, Zagazig, Egypt; ^5^ Department of Pathology and Clinical Pathology, Faculty of Veterinary Medicine, King Salman International University, Ras Sidr, Egypt; ^6^ Department of Pathology, Faculty of Veterinary Medicine, Zagazig University, Zagazig, Egypt; ^7^ Department of Biochemistry, College of Science, University of Jeddah, Jeddah, Saudi Arabia; ^8^ Department of Pharmaceutical Sciences, College of Pharmacy, Princess Nourah Bint Abdulrahman University, Riyadh, Saudi Arabia; ^9^ Department of Clinical Laboratory Sciences, Turabah University College, Taif University, Taif, Saudi Arabia; ^10^ Department of Pharmacology and Toxicology, College of Pharmacy, Jazan University, Jazan, Saudi Arabia; ^11^ Department of Medical Histology and Cell Biology, Faculty of Medicine, Zagazig University, Zagazig, Egypt; ^12^ College of Medicine, University of Ha’il, Ha’il, Saudi Arabia; ^13^ Department of Medical Microbiology and Immunology, Faculty of Medicine, Kafrelsheikh University, Kafr ElSheikh, Egypt

**Keywords:** pyrethroids, proinflammatory cytokines, CD56, caspase-3, mRNA expression, anemia, ROS, MDA

## Abstract

**Introduction:**

Conflict reports exist on the impact of pyrethroid insecticides on immune function and the probable underlying mechanisms.

**Methods:**

This study evaluated the effect of an extensively used pyrethroid insecticide, fenpropathrin (FTN) (15 mg/kg b.wt), on the innate and humoral immune components, blood cells, splenic oxidative status, and mRNA expression of CD3, CD20, CD56, CD8, CD4, IL-6, TNF-α, and Caspase3 in a 60-day trial in rats. Besides, the possible defensive effect of curcumin-loaded chitosan nanoparticle (CML-CNP) (50 mg/kg b.wt) was evaluated.

**Results:**

FTN exposure resulted in hypochromic normocytic anemia, thrombocytosis, leukocytosis, and lymphopenia. Besides, a significant reduction in IgG, not IgM, but increased C3 serum levels was evident in the FTN-exposed rats. Moreover, their splenic tissues displayed a substantial increase in the ROS, MDA, IL-6, and IL-1β content, altered splenic histology, and reduced GPX, GSH, and GSH/GSSG. Furthermore, a substantial upregulation of mRNA expression of splenic CD20, CD56, CD8, CD4, CD3, IL-6, and TNF-α, but downregulation of CD8 was detected in FTN-exposed rats. FTN exposure significantly upregulated splenic Caspase-3 and increased its immunohistochemical expression, along with elevated TNF-α immunoexpression. However, the alterations in immune function, splenic antioxidant status, blood cell populations, and immune-related gene expression were notably restored in the FTN + CML-CNP-treated group.

**Conclusion:**

The findings of this study highlighted the immunosuppressive effects of FTN and suggested the involvement of many CD cell markers as a potential underlying mechanism. Additionally, the results demonstrated the effectiveness of CML-CNP in mitigating pollutant-induced immune disorders.

## Graphical Abstract

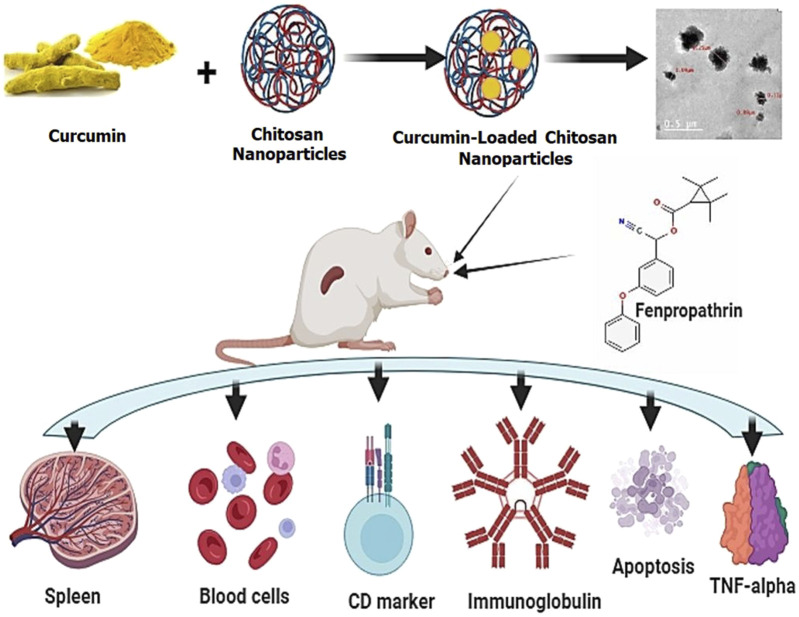

## 1 Introduction

Pyrethroids have long been known for their insecticidal properties (Iyadurai et al., 2023). Still, new reports on their possible use as adjuvants in antiviral and anticancer medicines have interested the public in learning more about their safety and immunotoxicity (Sharma et al., 2018). Because type II pyrethroids can easily bind to receptors on the B and T lymphocytes surface, there is debate over their immunosuppressive function and the danger of hypersensitivity induction (Jaremek and Nieradko-Iwanicka, 2020). In the recent *in silico* immunotoxic assessment by Kumar et al. (2018), Type II pyrethroids showed good interactions with immune cell proteins, which may be associated with several pathways like no change, autoimmune disorders, decreased immune response, and hypersensitive reactions.

Fenpropathrin (FTN) is a widely used pyrethroid pesticide in agriculture to fight numerous mites and pests in fruits, vegetables, and Chinese herbal medicine (Xiang et al., 2022; Liu et al., 2023). It can be frequently detected in water, sediments, and food products (Heshmati et al., 2019; Yang et al., 2022; Yildirim and Ciftci, 2022). The principal insecticidal activity of FTN is neurotoxicity, which can disrupt the operation of the sodium ion channel that is not tetrodotoxin-sensitive (Saputra et al., 2023). For a long period, FTN was thought to be safe for usage with mammals. Nonetheless, numerous studies conducted in the past few years have demonstrated that FTN can cause harm to various bodily organs, including the testis, kidneys, brain, liver, and intestines (Mohamed et al., 2019; Abd-Elhakim et al., 2020a; Jaremek and Nieradko-Iwanicka, 2020; Abu Zeid et al., 2021; Xu et al., 2022; Alqahtani et al., 2023). In a study conducted by Jaremek and Nieradko-Iwanicka (2020), it was found that during sub-acute FTN poisoning, lymphocytes, endothelial cells, and mesangial cells produced significant quantities of the pro-inflammatory cytokine such as tumor necrosis factor-alpha (TNF-α), interleukin-6 (IL-6), and interleukin-1beta (IL-1β) despite the absence of signs of significant organ dysfunction. There is mounting evidence that pro-inflammatory mediators are the primary drivers of immunological dysfunction. This finding could help illuminate the seeming contradiction of immune suppression in patients exhibiting hyperinflammation symptoms (Morris et al., 2013; Sacdalan and Lucero, 2021). Evidence suggests that oxidative stress propagation and apoptosis contribute to the cytotoxic effects of FTN (Mohamed et al., 2019; Jebur et al., 2023). Nonetheless, the impact of FTN exposure on immune functions has not been fully understood.

Using plant-bioactive compounds to modulate immune function altered by environmental pollutants is now of utmost importance (Abd-Elhakim et al., 2018a). One of the natural phenolic compounds found in turmeric (*Curcuma longa* L.), a member of the Zingiberaceae family, is known as curcumin (CM) (Divyashree et al., 2023). Its antioxidant, anticancer, anti-inflammatory, antibacterial, and pleiotropic biological and pharmacological characteristics have piqued the interest of researchers (Saber et al., 2019; Abd-Elhakim et al., 2021a; Abd-Elhakim et al., 2021b; Abd-Elhakim et al., 2022). Furthermore, CM has been shown in the latest study of Mehany et al. (2023) to protect against immunological diseases caused by environmental pollutants. CM has limited therapeutic application despite its many pharmacological activities because of its rapid hydrolysis, low oral bioavailability, and instability at physiological pH (Divyashree et al., 2023). One common way that hydrophobic and hydrophilic medications are delivered is by chitosan, a biocompatible and biodegradable polymer (Abd El-Hakim et al., 2020). Currently, nanotechnology has rapidly advanced due to the extensive utilization of nanoparticles (NPs) in many industrial and medical applications (Alsaba et al., 2020; Mhlanga et al., 2024). The utilization of nanomaterials has facilitated the development of sophisticated nano-based nutraceuticals characterized by improved solubility, bioavailability, encapsulation efficacy, consistency, prolonged therapeutic targeting, safety, and superior pharmacological activities (Manocha et al., 2022). Chitosan nanoparticles (CNP) represent a significant advancement in nanomaterial synthesis, serving as a reducing, stabilizing, and capping agent to enhance nanoformulation properties for applications in medicine and environmental science (Frank et al., 2020; Ben Amor et al., 2024). Thus, to improve the bioavailability and efficiency of CM, researchers are encasing it in CNP to make it more stable and more soluble and prolong its release (Asif et al., 2023). Because of its superior drug transport properties, chitosan is ideal for the oral administration of CM (Saheb et al., 2019; Ali et al., 2022). Khan et al. (2016) reported that CML-CNP exhibited improved cellular uptake, bioavailability, and stability in cervical cancer cells. Additionally, compared to free CM, CML-CNP showed a markedly higher uptake rate in the SiHa cells (Khan et al., 2018). A regulated and prolonged release pattern was also seen in the *in vitro* study of CML-CNP absorption by two cell lines: mouse mononuclear macrophage leukemia cells and human umbilical vein endothelial cells (Li et al., 2019). A latest study by Ali et al. (2022) found that when rats were subjected to cold stress, the oral dosing of CML-CNP had enhanced gastroprotective and neuroprotective effects than the conventional oral CM. Furthermore, CML-CNP exhibited immune stimulatory activity in aquatic organisms (Bhoopathy et al., 2021), but there is scarce information on its immunomodulation in mammals.

To our knowledge, no research has been published on the potential protective benefits of CML-CNP against FTN-induced immunological disruptions in rats. Hence, this study has two objectives. The initial goal is to explore the possible causal mechanisms of FTN-related immunological problems, emphasizing cytokine control. Second, to investigate the efficacy of CML-CNP in reducing FTN-induced immune changes. Biochemical, histological, molecular, and immunohistochemical research were carried out in rats orally administered FTN and/or CML-CNP over 60 days to achieve these goals.

## 2 Material and methods

### 2.1 Tested compounds

Sigma Aldrich Co. (St. Louis, Mo, United States) supplied the CM (C_21_H_20_O_6_, CAS: 458-37-37) and chitosan (CAS No. 9012-76-4). Additionally, commercially, we obtained the FTN emulsion concentration (C_22_H_23_NO_3_, Danitol comprises 20% FTN and 80% other components) from Sumitomo Chemical Co., Ltd. (Tokyo, Japan). The corn oil used to make the stock FTN solution was sourced from Arma Food Industries on the 10th of Ramadan, Egypt. Furthermore, all the chemicals and compounds utilized had the highest possible purity.

### 2.2 CML-CNP preparation and characterization

According to Kunjachan et al. (2010), the conventional method of ionic gelation synthesis was adopted for CML-CNP production. After combining 1% aqueous acetic acid with 0.5% chitosan and stirring at room temperature, a 0.5% chitosan solution was formed. After adding 1 mL of Tween 80 to the chitosan solution, the mixture was stirred for 30 min to ensure smoothness. We used a homogenizer (IKA®-Werke GmbH and Co. KG, Staufen, Germany) to mix the chitosan solution for 10 min at 3,320 × g. Then, we gently added the CMN solution dissolved in ethanol, being sure to keep the ratio of chitosan to CM at 1:1. Sodium tripolyphosphate (Na_5_P_3_O_10_; CAS No. 7,758-29-4, Sigma Aldrich Co., St. Louis, Mo, United States; 0.66% of chitosan weight) was dissolved in pure water and added drop by drop to the chitosan mixture while continuously stirring for 30 min. The solution’s turbidity confirmed the presence of CML-CNPs. The suspension was gone for an hour before being cooled to 4°C and kept until required. Many measures have been implemented throughout CML-CNP synthesis to prevent endotoxin contamination. These include using analytical-grade chemicals, sanitized glassware, and high-purity water. The shape and size of the CML-CNP particles were assessed by a high-resolution transmission electron microscope (HR-TEM, JEM-2100, JEOL, Tokyo, Japan). An instrument known as a Zeta Sizer (Nano-ZS, Zetasizer Ver, Malvern, United Kingdom) was used to determine the zeta potential of the synthesized CML-CNP. We used a Fourier transform infrared spectrometer (ALPHA II, Compact FT-IR spectrometer, Bruker, Germany) in the 400–4,000 cm^1^ range to identify the surface functional groups of CML-CNP. CML-CNP features were proven in our recent study (Mohamed et al., 2023).

### 2.3 Experimental animals and scheme

Forty male Sprague Dawley rats, aged three months and weighing 165 ± 0.37 g, were obtained from the Laboratory Animal Housing Unit at the Faculty of Veterinary Medicine, Zagazig University. The rats were kept in stainless steel cages with free access to food and water in a well-ventilated environment featuring a 12-h light/dark cycle. Prior to the experimental work described herein, they were retained in the laboratory for two weeks. The rats were randomly allocated into four groups, comprising ten rat each. The sample size was calculated using Resource equation method (Arifin and Zahiruddin, 2017).1 mL of corn oil was administered orally to each rat in the control group. The group that received CML-CNP was orally given 50 mg/kg b.wt of CML-CNP. Compared to the same dose of conventional CM, the anti-inflammatory and antioxidant effects of 100 mg CML-CNP/kg b.wt given orally to rats for 14 days were much more pronounced in the prior work by Ali et al. (2022). As the current experiment was conducted for a longer time (60 days), the current study evaluated CML-CNP at the lower dose (50 mg/kg b.wt). In line with the study of Mohamed et al. (2019), the FTN group was orally given 15 mg of FTN (dissolved in corn oil)/kg b. wt. The FTN + CML-CNP groups orally received the FTN and CML-CNP at the doses declared above with a one-h interval during the 60-day.

### 2.4 Blood and tissue sampling

Rats were administered an intraperitoneal injection of 50 mg/kg b. wt of xylazine and 5 mg/kg b. wt ketamine hydrochloride, after which blood was extracted from the retro-orbital venous plexus of each rat and divided into two samples. The initial blood specimen was placed into an EDTA tube for hematological analysis. Non-heparinized tubes were utilized to collect the second blood sample. Following a 20-min coagulation period, the blood was centrifuged at 664 × g for 10 min to separate serum, which was subsequently stored at −20°C until biochemical analysis. Rats were decapitated following the collection of blood samples to obtain the spleen. We categorized the necropsied splenic tissue into three sets. The initial group underwent flash freezing at −80°C for gene expression analysis. The second set of samples was homogenized using a Potter-Elvehjem tissue homogenizer (Thomas Scientific, Swedesboro, NJ, United States) with cold potassium chloride and centrifuged at 4°C for 10 min to obtain the homogenate. Quantification of antioxidant, pro-inflammatory, and apoptotic factors was then performed using the supernatants. The remaining splenic tissues were prepared for histology and immunohistochemistry using a 10% buffered formalin solution.

### 2.5 Evaluation of hematological parameters

The HemaScreen 18 automated hematology analyzer (Hospitex Diagnostics, Sesto Fiorentino, Italy) was employed to measure the following parameters, as per the method outlined by Feldman et al. (2000): hemoglobin (Hb), packed cell volume (PCV), total red blood cells (RBCs), mean cell volume (MCV), mean corpuscular hemoglobin concentration (MCHC), total leukocytes, lymphocytes, granulocytes, and MID cells. The automated analyzer was employed to conduct the total leukocyte counts, while the manual procedure proposed by Dacie and Lewis (1991) was employed to conduct the differential leukocyte counts.

### 2.6 Analysis of serum immunological indices

Commercial ELISA kits were used to estimate serum concentrations of immunoglobulin M (IgM). The kits were obtained from MyBioSource (San Diego, CA, United States). The sensitivity of the assays was 4.69 ng/mL, and the detection range was 7.81–500 ng/mL. The procedures were followed precisely as instructed by the manufacturers. Commercial ELISA kits from Invitrogen were used to measure serum immunoglobulin G (IgG) levels. These kits have a detection range of 1.6–100 ng/mL and a sensitivity of 1.6 ng/mL. To measure complement 3 (C3) serum levels, we used rat EISA kits manufactured by Crystal Chem (IL, United States), which have a sensitivity of 4.4 ng/mL and a detection range of 12.5–800 ng/mL.

### 2.7 Evaluation of splenic oxidative stress and inflammatory markers

Splenic homogenates were tested for reactive oxygen species (ROS) content using a Nova Lifetech assay (Catalog Number E1924r; detection range: 15.6 pg/mL −1,000 pg/mL; Hong Kong, China). To quantity the glutathione peroxidase (GPX) levels, an ELISA kit (Cat. No. MBS1600242) from MyBioSource (San Diego, United States) was utilized. The detection range of the kit was 0.5–200 ng/mL, and its sensitivity was 0.24 ng/mL. Testing kits of Bio-diagnostic Co. (Cat. No. GR 25 11, Dokki, Giza, Egypt) were used to determine the levels of reduced glutathione (GSH). The GSH/GSSG ratio was measured using a BioAssay Systems EnzyChromTM GSH/GSSG Assay Kit (EGTT-100) in Hayward, CA, United States. Nevertheless, the homogenized splenic tissue was analyzed for malondialdehyde (MDA) using biodiagnostic kits from Dokki, Giza, Egypt (CAT. No. MD 25 29). Additionally, inflammatory markers were assessed in the splenic homogenates. IL1β concentration was estimated using Cusabio (Houston, TX, United States) rat IL-1β ELISA kits (Catalog Number CSB-E08055r, Sensitivity: 15.6 pg/mL, and detection range: 62.5–4,000 pg/mL). Interleukin-6 (IL-6) levels were evaluated by MyBioSource (San Diego, United States) ELISA kits (Cat. No. MBS2020158, Sensitivity: <3.3 pg/mL, and detection range: 7.8–500 pg/mL).

### 2.8 Gene expression

Through RNA extraction, the expression of genes associated with pro-inflammatory cytokines and cluster of differentiation (CD) cells, including CD56, CD20, CD8, CD4, CD3, IL-1β, TNF-α, and IL-6, was assessed in the splenic tissues of rats in all experimental groups. Primer in Table 1 were provided by Metabion (Germany). To obtain the final values for each sample, the “ΔΔCt” method was used in line with the technique outlined by Yuan et al. (2006).

**TABLE 1:** Primers sequences, accession number, and product size for the quantitative RT-PCR for the analyzed genes in the hepatic tissue.

Target gene	Forward primer	Reverse primer	bp	Accession no.
GAPDH	GCA​TCT​TCT​TGT​GCA​GTG​CC	TAC​GGC​CAA​ATC​CGT​TCA​CA	74	NM_017008.4
CD3	AAA​GGT​TTG​GCT​GGC​CTC​TT	GCC​ATC​TCC​TTG​GCT​GTC​AT	108	NM_001077646.2
CD8	ACT​CAC​GGA​GTG​TGC​TGA​AG	CAG​TCA​TGC​TGC​CCT​ACC​AA	137	NM_031539.2
CD4	AGA​AAG​GAC​TGG​CCA​GAG​AC	CTG​AAA​GAG​AAG​CCT​CGG​CA	73	NM_012705.1
CD20	CCA​GCT​GAT​CTC​AGC​AGT​GAA	TTT​TGA​GCA​GGT​TGC​ATG​GC	161	NM_001399452.1
CD56	ACA​AGG​CTG​AGT​GGA​AGT​CG	CGG​ACT​GGC​TGT​GTC​TTG​AA	199	NM_001395707.1
Casp-3	GAG​ACA​GAC​AGT​GGA​ACT​GAC​GAT​G	GGC​GCA​AAG​TGA​CTG​GAT​GA	147	NM_012922.2
IL-6	ATA​TGT​TCT​CAG​GGA​GAT​CTT​GGA​A	GTG​CAT​CAT​CGC​TGT​TCA​TAC​A	80	NM_012589.2
TNF-α	GGC​TTT​CGG​AAC​TCA​CTG​GA	GGG​AAC​AGT​CTG​GGA​AGC​TC	164	NM_012675.3

GAPDH, glyceraldehyde-3-phosphate dehydrogenase; TNF-α, tumor necrosis factor alpha; CD, cluster of differentiation; IL-6, interleukin 6 nucleotide-binding domain.

### 2.9 Histopathological assessment

For histopathology, the posterior portion of the spleen of each animal was sampled in line with the guides for organ collection and trimming in rats (Ruehl-Fehlert et al., 2003); a transverse section is made at the organ’s maximum extension to confirm the existence of all relevant histological structures of the white pulp, marginal zone, and follicles. The splenic tissue specimens were promptly fixed in 10% neutral buffered formalin for 24 h and thoroughly washed in distilled water, a series of ethanol dehydration steps, xylene clearing, and paraffin impregnation and embedding. The embedded tissues were sectioned at a thickness of 5 microns and stained with Harris’s Hematoxylin and Eosin Y stains following the instructions of Suvarna et al. (2018) and examined microscopically. For precise quantification of the microscopic changes in all groups, if any, a multiparametric quantitative grading system for histopathology was used, and the results were indicated as mean ± SE. Five 10× non-overlapped microscopic fields were randomly selected for each rat and were microphotographed using an AmScope microscope digital camera (MU1803-HS) (United Scope LLC, CA, United States). These photomicrographs (50 per group) were interpreted, and the encountered histological alterations were statistically analyzed. The lesion frequency (the number of times the lesion occurs) and severity (distribution) were ultimately quantified. The lesion frequency was calculated using the formula: frequency = Nlesion ÷ Ntotal × 100, where Nlesion is the total number of images that exhibited a specific lesion, and Ntotal is the total number of images in the group (50), while a Four-point scale was assigned to quantify the lesion severity: 0 indicated no lesion at all, 1, focal distribution, 2, multifocal distribution, and 3, diffuse distribution.

### 2.10 Immunohistochemical investigation of the caspase-3 and TNF-α biomarkers

For each rat, two splenic tissue sections, each five microns thick, were obtained and immunostained following the avidin-biotin-peroxidase complex protocol established by Hsu et al. (1981). The first section was incubated with rabbit monoclonal anti-caspase-3 primary antibody [EPR18297] (ab184787, Abcam, Inc.) at 1/1,000 dilution for labeling the pro and active CASP3, while the second section was incubated with mouse monoclonal anti-TNF-α primary antibody [TNFA/1,172] (ab220210, Abcam, Inc.) at 4 μg/mL dilution. The immune complex deposits were visualized by 3,3′-Diaminobenzidine (DAB) chromogen, and the nuclei were counterstained by Harris hematoxylin. Next, for quantification of the immunoexpression of both biomarkers, for each rat, five 40×, fixed size, non-overlapped microscopic fields, selected at random were microphotographed using the AmScope microscope digital camera (MU1803-HS) (United Scope LLC, CA, United States), at the same exposure time, same objective lens, and same lighting. These photomicrographs (50 per group) were analyzed using the open-source ImageJ software via the color deconvolution plugin as indicated by Schneider et al. (2012), determining the percentages of the brown color area fraction to the total areas of the images, and the data were presented as mean ± SE.

### 2.11 Data analysis

The Kolmogorov-Smirnov test was adopted to assess the normality of the data distribution, whereas Levene’s test was used to evaluate the homogeneity of variances. A one-way analysis of variance (ANOVA) was employed to statistically assess the variation among groups using IBM SPSS Statistics, version 21 (IBM; Armonk, New York, United States) (Spss, 2011). The assumptions of normality were then met by employing Tukey’s multiple range *post hoc* test for pairwise comparisons. Histopathological scores were analyzed using a Kruskal–Wallis test, which was subsequently followed by Dunn’s multiple comparisons test. The results are presented as means ± SE. Significant variations in the means were recorded at the p < 0.05 level.

## 3 Results

### 3.1 Effects on hematological parameters

The impact of CML-CNP co-treatment on the hematological parameters of FTN-exposed rats was illustrated in Table 2. Compared to the control rats, the FTN-exposed rats exhibited a significant decrease in RBC count (p < 0.001), Hb (p = 0.002), and PCV (p < 0.001). Conversely, a significant elevation in MCHC (p < 0.001), MCH (p < 0.001), and platelet count (p = 0.037) was detected in the FTN-exposed rats relative to the control group. FTN + CML-CNP-treated rats had a significantly elevated RBC count (p = 0.045), Hb content (p = 0.042), and PCV % (p = 0.017), while demonstrating decreased MCH (p = 0.004) and MCHC (p = 0.023) in comparison to FTN-exposed animals. A significant increase in platelet count (p = 0.037) was obvious in the FTN + CML-CNP-treated group relative to the control group. Moreover, Hb content, RBC count, and PCV % exhibited no significant differences between the FTN + CML-CNP-treated group and the control group. No significant alterations in MCV were found across the different experimental groups.

**TABLE 2:** Effect of curcumin loaded chitosan nanoparticles (CML-CNP) oral dosing on hematological indices of adult male Sprague Dawely rats exposed to fenpropathrin (FTN) for 60 days.

Estimated parameters	C	CML-CNP	FTN	CML-CNP+FTN
Erythrogram				
RBCs (10^6^/mm^3^)	6.43 ± 0.10	6.52 ± 0.50	4.24 ± 0.12***	5.36 ± 0.01^#^
Hb (g/dL)	16.27 ± 0.20	16.43 ± 0.42	14.40 ± 0.11**	15.57 ± 0.24^#^
PCV (%)	35.43 ± 0.87	35.50 ± 1.29	26.17 ± 0.73***	31.33 ± 1.09^#^
MCV(fl)	55.10 ± 0.97	55.06 ± 2.13	61.74 ± 0.86	58.43 ± 2.13
MCH (%)	25.31 ± 0.21	25.56 ± 1.20	34.07 ± 0.94***	29.03 ± 0.45*##
MCHC (%)	45.97 ± 0.60	46.37 ± 0.58	55.17 ± 1.27***	49.87 ± 1.57#
Platelets	330.00 ± 13.77	330.33 ± 16.53	412.33 ± 29.94*	410.33 ± 2.09*
Leukogram				
WBCs (10^3^/mm^3^)	7.03 ± 0.24	7.07 ± 0.10	12.87 ± 1.34***	7.13 ± 0.39^###^
Lymphocytes %	72.30 ± 0.96	71.97 ± 1.20	60.17 ± 0.23***	71.43 ± 0.85^###^
MID %	15.63 ± 0.48	15.73 ± 0.29	23.53 ± 1.01***	15.90 ± 0.14^###^
Granulocytes %	12.07 ± 0.51	12.30 ± 0.33	16.30 ± 0.47***	12.67 ± 0.30^###^

Means within same row carrying different superscripts are significant different at *p* < 0.05. Values shown are means ± SE. n = 10 group. *p < 0.05, **p < 0.01, and ***p < 0.001 vs control and ^#^p < 0.05, ^##^p < 0.01, and ^###^p < 0.001 vs. FTN. RBCs, red blood cells; Hb, hemoglobin; PCV, packed cell volume; MCV, mean corpuscular volume; MCH, mean corpuscular hemoglobin; MCHC, mean corpuscular hemoglobin concentration; WBC, white blood cells.

Concerning the leukogram alterations, a significant (p < 0.001) increase in WBCs, granulocytes, and MID cell count was found in FTN-exposed rats than the control rats (Table 2). On the contrary, a significant (p < 0.001) decrease in the lymphocyte count was more evident in the FTN-exposed group than in the control group. However, FTN + CML-CNP-treated rats had significantly (p < 0.001) lower WBCs, MID cell, and granulocytes count but a higher lymphocyte count than FTN-exposed rats.

### 3.2 Effects on innate immune components

**FIGURE 1:**
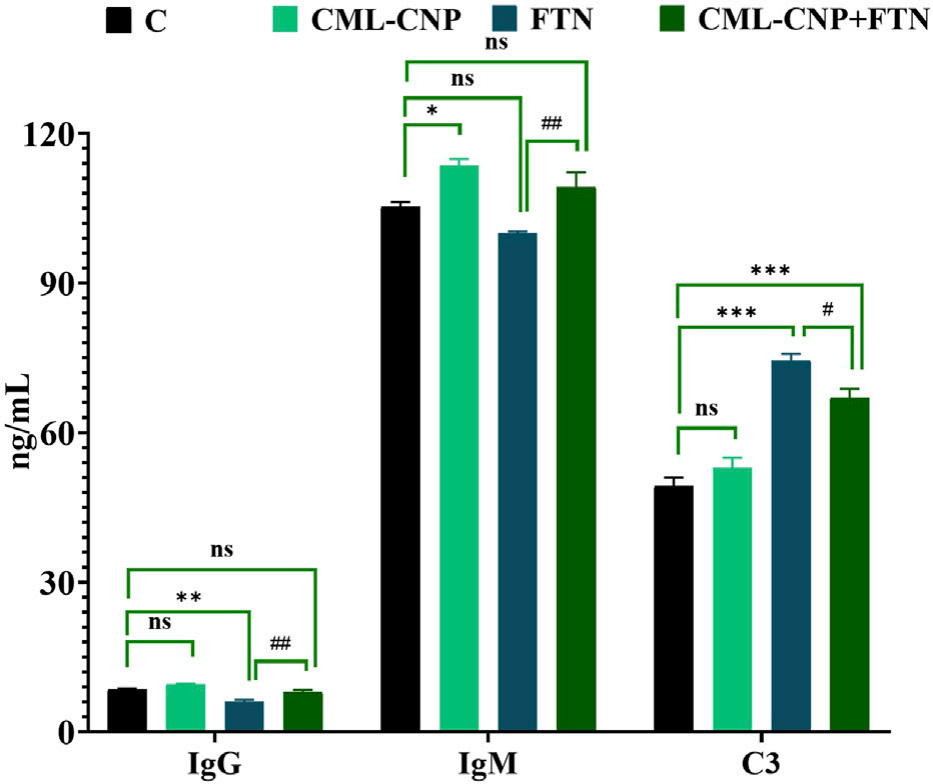
Effect of curcumin-loaded chitosan nanoparticles (CML-CNP) oral dosing on immunoglobulin G (IgG), immunoglobulin M (IgM), and complement 3 (C3) in the serum of adult male Sprague Dawley rats exposed to fenpropathrin (FTN) for 60 days. Bars represent the mean ± SE. n = 10. *p < 0.05, **p < 0.01, and ***p < 0.001 vs. control and #p < 0.05 and ##p < 0.01 vs. FTN.

As demonstrated in Figure 1, a significant (p = 0.02) increase in serum IgM was recorded in the CML-CNP-treated rats by 12% compared to the control rats. Conversely, the FTN-exposed rats exhibited a significant (p = 0.001) reduction in the serum IgG by 29% but a critical (p < 0.001) increment in C3 by 51% than the control group. Yet, FTN + CML-CNP-treated rats had a significantly higher IgM (p = 0.009) and IgG (p = 0.004) but a significantly (p = 0.04) lower C3 relative to FTN-exposed rats. Remarkably, IgG and C3 serum levels did not differ significantly between the FTN + CML-CNP-treated and control groups.

### 3.3 Effects on splenic oxidative status

**FIGURE 2:**
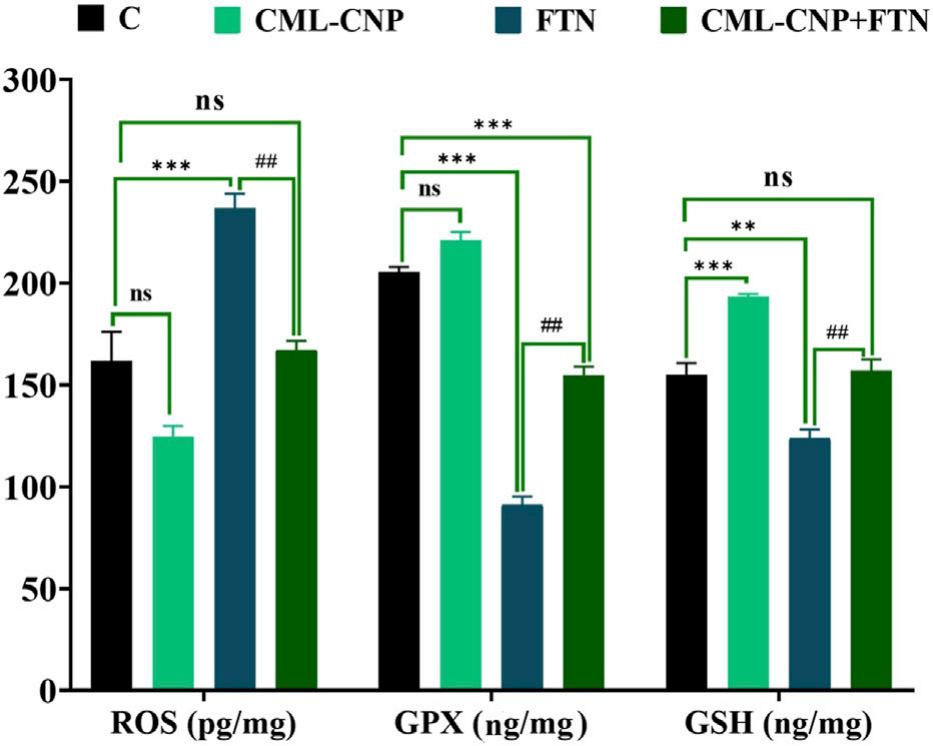
Effect of curcumin-loaded chitosan nanoparticles (CML-CNP) oral dosing on reactive oxygen species (ROS), glutathione peroxidase (GPX), and reduced glutathione (GSH) in the splenic tissues of adult male Sprague Dawley rats exposed to fenpropathrin (FTN) for 60 days. Bars represent the mean ± SE. n = 10. *p < 0.05, **p < 0.01, and ***p < 0.001 vs. control and #p < 0.05 and ##p < 0.01 vs. FTN.

**FIGURE 3:**
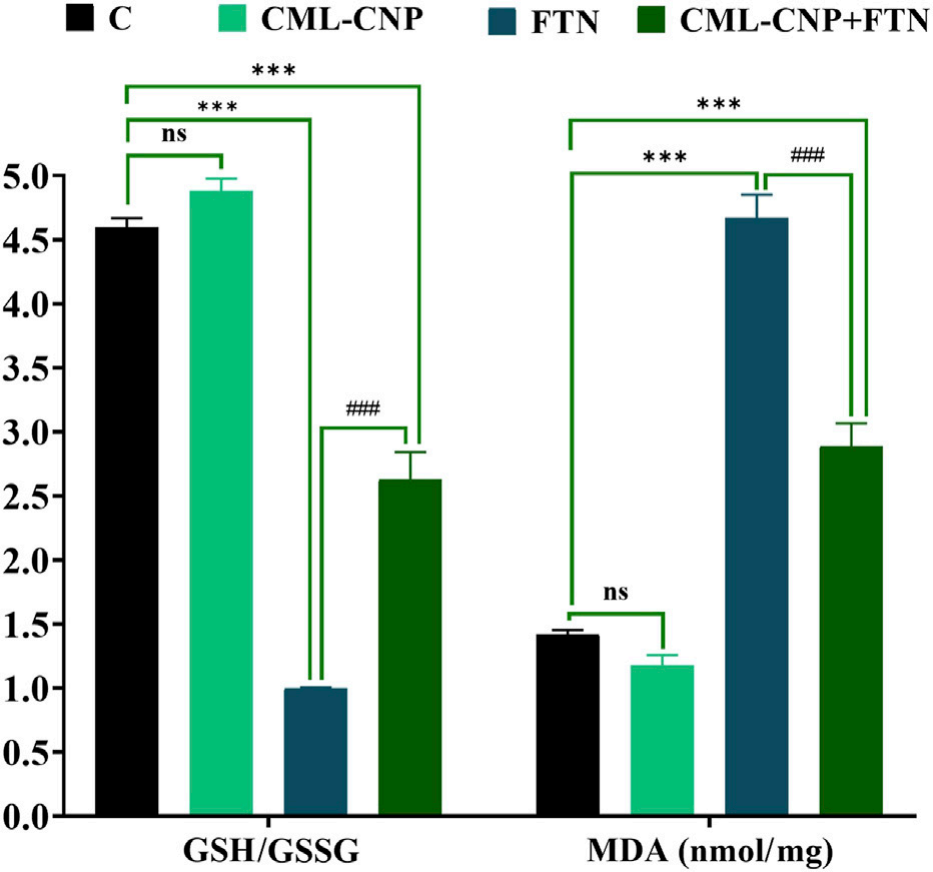
Effect of curcumin-loaded chitosan nanoparticles (CML-CNP) oral dosing on reduced glutathione/oxidized glutathione ratio (GSH/GSSG) and malondialdehyde (MDA) in the splenic tissues of adult male Sprague Dawley rats exposed to fenpropathrin (FTN) for 60 days. Bars represent the mean ± SE. n = 10. *p < 0.05, **p < 0.01, and ***p < 0.001 vs. control and #p < 0.05 and ##p < 0.01 vs. FTN.

As revealed in Figure 2, the spleen of the CML-CNP-treated rats had a significantly (p < 0.001) greater GSH content by 25% than the control one. Oppositely, relative to the control rats, a significant reduction of GSH (p = 0.002, 20%) and GPX (p < 0.001, 56%) levels, and GSH/GSSG (p < 0.001, 78%) but increment in the concentrations of ROS (p < 0.001, 46%) and MDA (p < 0.001, 229%) was recorded in the FTN-exposed rats (Figures 2, 3). Nonetheless, the FTN + CML-CNP-treated group demonstrated a significantly higher GSH/GSSG (p < 0.001) and GSH (p = 0.001) and GPX (p < 0.001) levels but a considerably lower MDA (p < 0.001) and ROS (p = 0.001) than the FTN-exposed rats. No significant changes in the ROS and GSH were detected between the FTN + CML-CNP-treated and control groups.

### 3.4 Changes in pro-inflammatory cytokines concentrations in splenic homogenate

**FIGURE 4:**
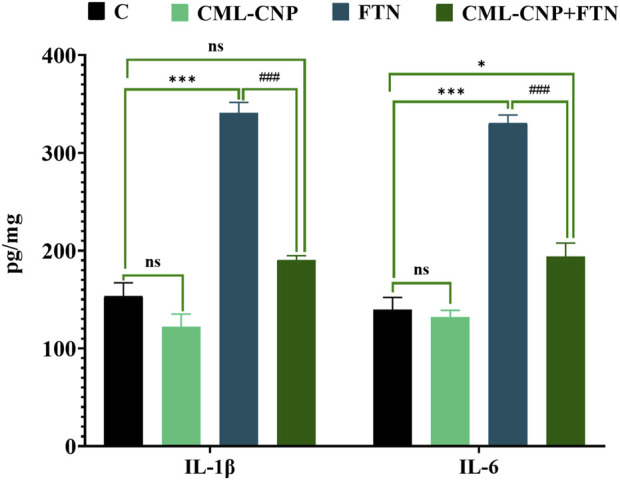
Effect of curcumin-loaded chitosan nanoparticles (CML-CNP) oral dosing on interleukin 1β (IL1β) and interleukin-6 (IL-6) in the splenic tissues of adult male Sprague Dawley rats exposed to fenpropathrin (FTN) for 60 days. Bars represent the mean ± SE. n = 10. *p < 0.05, **p < 0.01, and ***p < 0.001 vs. control and #p < 0.05 and ##p < 0.01 vs. FTN.

The FTN oral dosing significantly (p < 0.001) increased the splenic levels of IL-1β and IL-6 by 122% and 137%, respectively than the control group (Figure 4). However, FTN + CML-CNP-treated rats had significantly (p < 0.001) lower splenic levels of IL-1β and IL-6 than FTN-exposed rats. Notably, IL-1β splenic content did not change significantly between the FTN + CML-CNP-treated and control groups.

### 3.5 Histopathological findings

**FIGURE 5:**
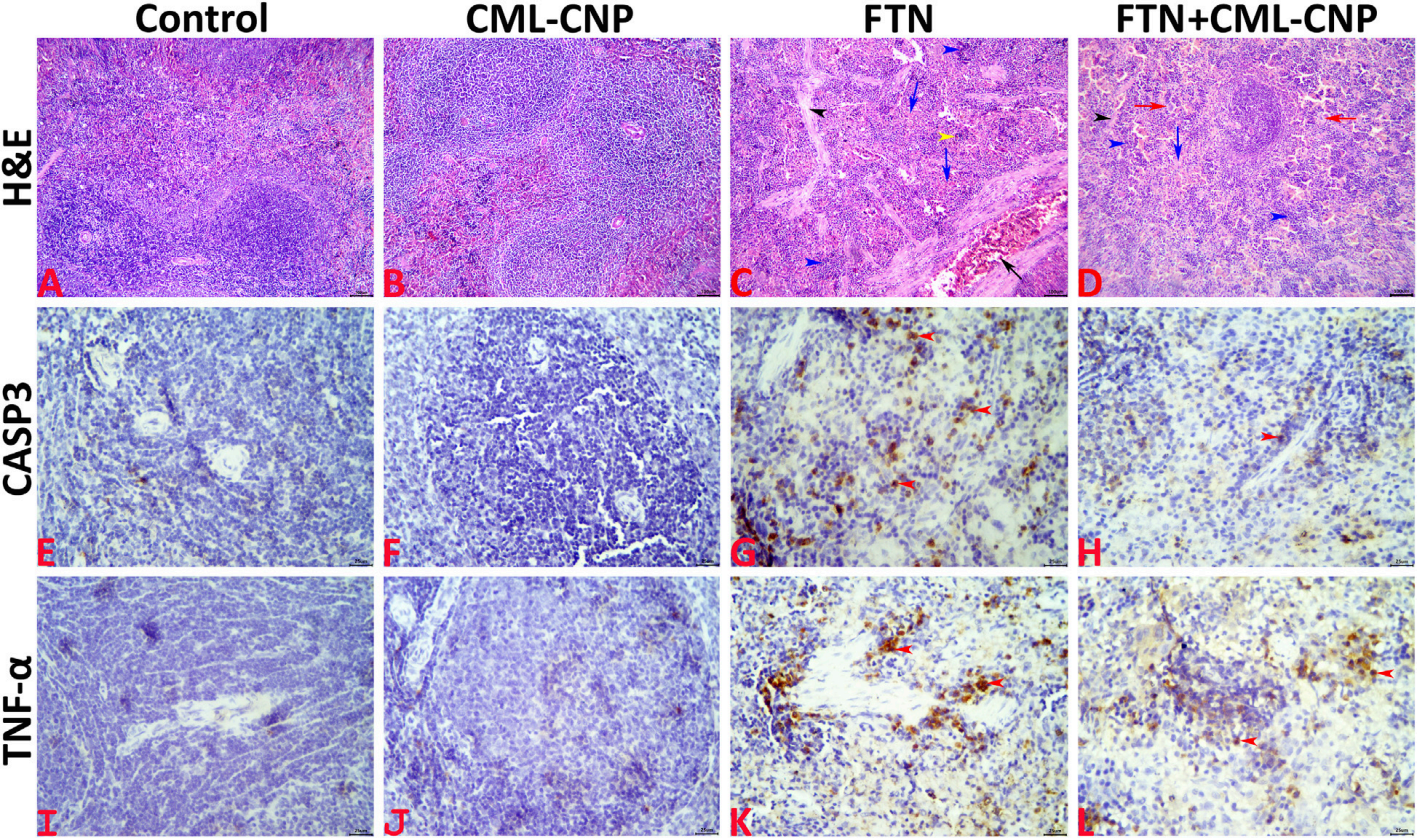
**(A–D)**: Representative photomicrograph of the H&E-stained splenic tissue sections showing normal histology in the control **(A)** and CML-CNP **(B)** groups. The spleen of the FTN group shows vascular congestion (black arrow), proliferated trabecular tissue (black arrowhead), extramedullary hemopoiesis for the erythroid (blue arrows), and lymphoid (blue arrowheads) elements, and hemosiderosis (yellow arrowhead) **(C)**. The spleen of the FTN + CML-CNP group shows normal trabecular content (black arrowhead), extramedullary hemopoiesis for the erythroid (blue arrow), and lymphoid (blue arrowheads) elements, and increased numbers of megakaryocytes (red arrows) **(D)**. The scale bars = 100 microns (10×). **(E–H)**: Representative photomicrograph of the CASP3-stained splenic tissue sections showing (1) almost negative expression in the control **(E)**, and CML-CNP **(F)** groups, (2) significant upregulation to the CASP3 expression (red arrowheads) in the FTN **(G)**, and FTN + CML-CNP **(H)** groups compared to the control and CML-CNP groups, and (3) significant downregulation to the CASP3 expression in the FTN + CML-CNP group compared to the FTN group. The scale bars = 25 microns (40×). **(I–L)**: Representative photomicrograph of the TNF-α-stained splenic tissue sections showing (1) almost negative expression in control **(I)**, and CML-CNP **(J)** groups, (2) significant upregulation to the TNF-α expression (red arrowheads) in the FTN **(K)**, and FTN + CML-CNP **(L)** groups compared to the control and CML-CNP groups, and (3) significant downregulation to the TNF-α expression in the FTN + CML-CNP group compared to the FTN group. The scale bars = 25 microns (40×).

All splenic tissue sections of the control and CML-CNP groups showed normal splenic histology with no histopathological alterations (Figures 5A, B). The splenic response to the oral FTN exposure involved splenic cellularity, architecture, and vasculature changes. Most examined sections exhibited notable vascular congestion, extramedullary hematopoiesis to the erythroid and lymphoid elements, and distorted architecture by increased trabecular connective tissue elements (Figure 5C). Additionally, endothelial hypertrophy, hemosiderosis, and increased numbers of megakaryocytes were evident. Compared to the FTN group, the splenic tissue sections of the FTN + CML-CNP group showed (1) a significant reduction in the connective tissue elements, hemosiderosis, vascular congestions, and endothelial hypertrophy, (2) a significant increase in the numbers of megakaryocytes, and (3) no differences in the extramedullary hematopoiesis of the erythroid and lymphoid elements (Figure 5D). The overall splenic lesion scoring in all experimental groups was summarized in Table 3.

**TABLE 3:** Effect of curcumin loaded chitosan nanoparticles (CML-CNP) oral dosing on the splenic histology and immunoexpression of the CASP3 and TNF-α of adult male Sprague Dawely rats exposed to fenpropathrin (FTN) for 60 days.

Estimated parameters	C		CML-CNP		FTN		CML-CNP+FTN	
Lesions	S	F	S	F	S	F	S	F
Erythroid extra- medullary hematopoiesis	0.00 ± 0.00	0.00 ± 0.00	0.00 ± 0.00	0.00 ± 0.00	1.50*** ± 0.27	15.00*** ± 2.69	0.70^#^ ± 0.26	7.00^#^ ± 2.60
Lymphoid extra- medullary hematopoiesis	0.00 ± 0.00	0.00 ± 0.00	0.00 ± 0.00	0.00 ± 0.00	1.50*** ± 0.22	14.00*** ± 2.21	0.40^###^ ± 0.16	4.00^###^ ± 1.63
Lymphoid depletion	0.00 ± 0.00	0.00 ± 0.00	0.00 ± 0.00	0.00 ± 0.00	0.20 ± 0.13	2.00 ± 0.99	0.20 ± 0.13	2.00 ± 0.82
Increased connective tissue elements	0.00 ± 0.00	0.00 ± 0.00	0.00 ± 0.00	1.00 ± 1.00	0.50** ± 0.17	6.00* ± 2.21	0.10^#^ ± 0.10	1.00^#^ ± 1.00
Vascular congestion	0.00 ± 0.00	0.00 ± 0.00	0.00 ± 0.00	0.00 ± 0.00	1.70*** ± 0.21	15.00*** ± 2.24	0.50^###^ ± 0.17	5.00^###^ ± 1.67
Increased numbers of megakaryocytes	0.00 ± 0.00	0.00 ± 0.00	0.00 ± 0.00	0.00 ± 0.00	0.30 ± 0.15	3.00 ± 1.53	0.60^*^ ± 0.22	8.00^*^ ± 2.91
Endothelial hypertrophy	0.00 ± 0.00	0.00 ± 0.00	0.00 ± 0.00	0.00 ± 0.00	1.00*** ± 0.00	18.00*** ± 4.10	0.60^***##^ ± 0.16	8.00^#^ ± 1.15
Hemosiderosis	0.00 ± 0.00	0.00 ± 0.00	0.00 ± 0.00	0.00 ± 0.00	0.70*** ± 0.15	7.00*** ± 1.03	0.40 ± 0.16	4.00^**#^ ± 1.00
Immunostained area fraction								
CASP3	0.54 ± 0.22		0.32 ± 0.21		6.67*** ± 0.92		2.66^*###^ ± 0.30	
TNF-α	0.21 ± 0.08		0.14 ± 0.06		6.01*** ± 0.75		2.24^**###^ ± 0.27	

S, severity and F, frequency. Means within same row carrying different superscripts are significant different at p < 0.05. Values shown are means ± SE. n = 10 group. *P < 0.05, **p < 0.01, and ***p < 0.001 vs control and ^#^p < 0.05, ^##^p < 0.01, and ^###^p < 0.001 vs. FTN.

### 3.6 Immunohistochemical findings

Representative microphotographs for the CASP3 and TNF-α immunostained splenic tissue sections were shown in Figures 5E–H, I–L, respectively. The digitalized-image analysis and scoring of the positively stained area fractions of the CASP3 and TNF-α antigens declared the following (1) almost there was a negative expression of both CASP3 and TNF-α biomarkers in the splenic tissue sections of the control, and CML-CNP groups, (2) there was significant upregulation of the CASP3 and TNF-α expressions in the splenic tissue sections of the FTN, and FTN + CML-CNP groups compared to either the control or the CML-CNP groups, and (3) there was significant downregulation of the CASP3, and TNF-α expressions in the splenic tissue sections of the FTN + CML-CNP group compared to the FTN group. For simplicity, the numerical values (mean ± SE) of the immunostained area fractions of the CASP3 and TNF-α among all groups were presented in Table 3.

### 3.7 Changes in the pro-inflammatory cytokines, cluster of differentiation (CD), and apoptotic-related genes mRNA expression

**FIGURE 6:**
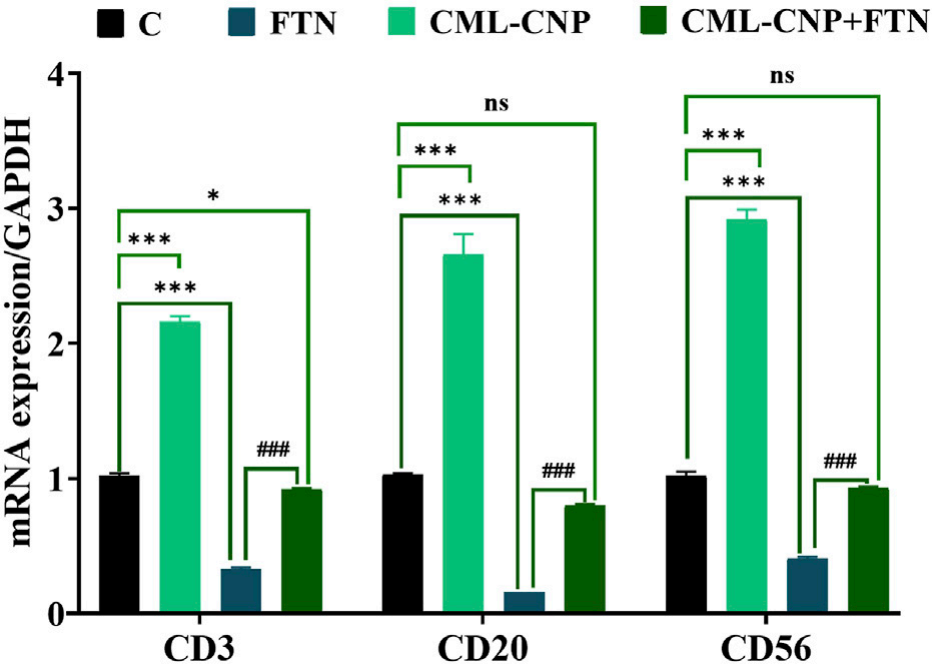
Effect of curcumin-loaded chitosan nanoparticles (CML-CNP) oral dosing on mRNA CD3, CD20, and CD56 expression in the splenic tissues of adult male Sprague Dawley rats exposed to fenpropathrin (FTN) for 60 days. Bars represent the mean ± SE. n = 10. *p < 0.05, **p < 0.01, and ***p < 0.001 vs. control and #p < 0.05 and ##p < 0.01 vs. FTN.

**FIGURE 7:**
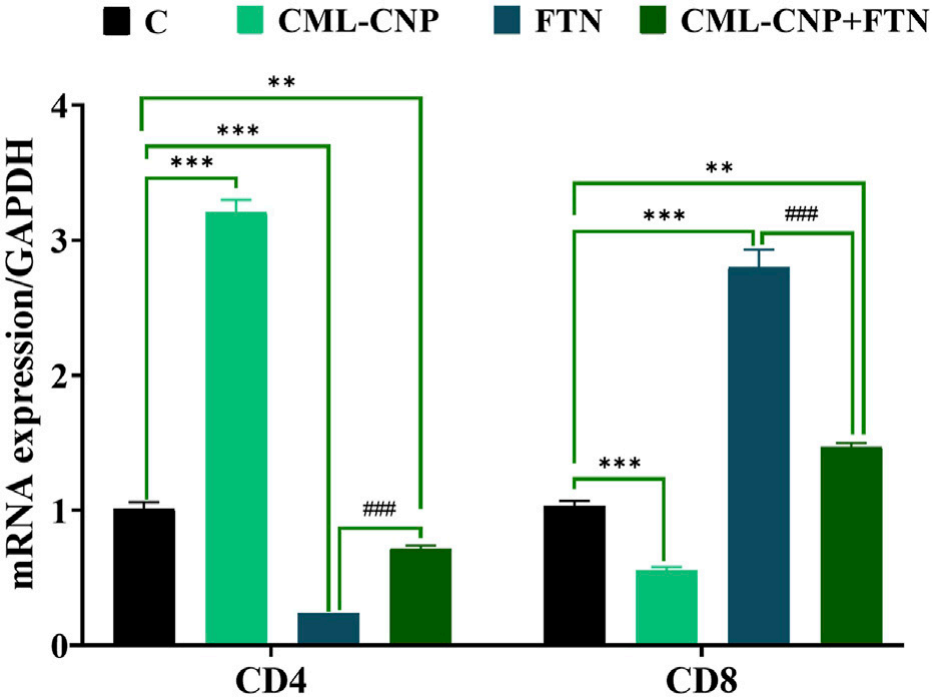
Effect of curcumin-loaded chitosan nanoparticles (CML-CNP) oral dosing on mRNA CD4 and CD8 expression in the splenic tissues of adult male Sprague Dawley rats exposed to fenpropathrin (FTN) for 60 days. Bars represent the mean ± SE. n = 10. *p < 0.05, **p < 0.01, and ***p < 0.001 vs. control and #p < 0.05 and ##P < 0.01 vs. FTN.

**FIGURE 8:**
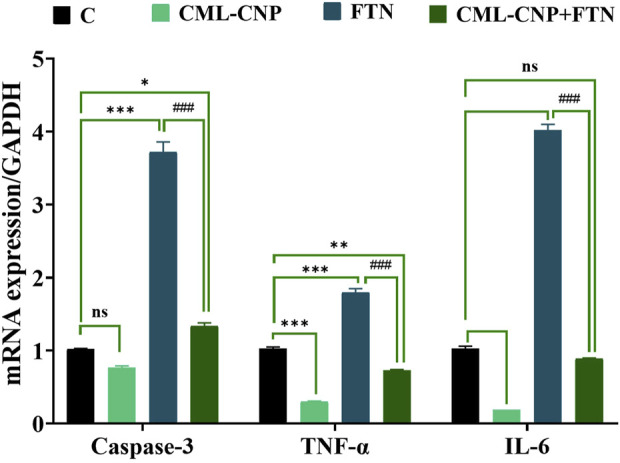
Effect of curcumin-loaded chitosan nanoparticles (CML-CNP) oral dosing on mRNA Caspase-3, TNF-α, and IL-6 expression in the splenic tissues of adult male Sprague Dawley rats exposed to fenpropathrin (FTN) for 60 days. Bars represent the mean ± SE. n = 10. *p < 0.05, **p < 0.01, and ***p < 0.001 vs. control and #p < 0.05 and ##p < 0.01 vs. FTN.

As displayed in Figures 6–8, a significant (p < 0.001) downregulation of CD3, CD20, CD56, and CD4 but a significant upregulation of CD8, Caspase3, IL-6, and TNF-α was found in the splenic tissues of the FTN-exposed rats than the control rats. Nonetheless, the FTN + CML-CNP-treated group demonstrated a significantly higher CD3, CD20, CD56, and CD4 mRNA expression but a considerably lower CD8, Caspase3, IL-6, and TNF-α relative to FTN-exposed rats. Remarkably, splenic CD20, CD56, and IL-6 mRNA expression did not change significantly between the FTN + CML-CNP-treated and control groups.

## 4 Discussion

Until now, little is known about the immune status alterations at the exposure to low levels of pyrethroid FTN for a long duration. For this reason, a multi-parameter analysis of immune constituents that are essential in immune defense and regulation was accomplished in the current study. It has been demonstrated that the synthetic pyrethroid FTN has profound effects on the immunocompetent blood cells in terms of obvious leukocytosis, lymphopenia, granulocytosis, and increased MID cells count. Comparably, leukocytosis was associated with exposure to other types of pyrethroids, including cypermethrin (Khan et al., 2009) and deltamethrin (Pimpão et al., 2007). This could be due to the shifting of the leukocytic pool from the spleen to the peripheral circulation (Haratym-Maj, 2002). Leukocytosis can also occur chronically in response to an inflammatory stressor/cytokine cascade (Rice and Jung, 2018). Moreover, *in vitro* cell tests with human lymphocytes showed that numerous pyrethroids suppress lymphocyte proliferation (Diel et al., 2003; Hadnagy et al., 2003). At the same time, lymphocytopenia and granulocytosis are reflected in decreased lymphocyte transformation to various antigens and impaired neutrophil chemotaxis is manifested by immunosuppression (Tsuchida et al., 2004). Herein, a considerable increase in serum C3 level was detected in the FTN-exposed rats. Moreover, a significant increase in the splenic pro-inflammatory cytokines levels, including IL-6 and IL-1β concomitant with a remarkable upregulation of TNF-α and IL-6 genes and increased TNF-α immunoexpression was evident following FTN oral dosing for 60 days. In this line, multiple proteolytic enzymes found in inflammatory exudates, including lysosomal enzymes and neutrophil elastase, have been demonstrated to activate C3 (Markiewski and Lambris, 2007). Pro-inflammatory cytokines like TNFα are crucial in the immunological and inflammatory response, control cell proliferation, and release chemicals that modulate the immune response and cell differentiation (Jaremek and Nieradko-Iwanicka, 2020). Yet, prolonged elevation of proinflammatory cytokines can lead to chronic inflammation, which may eventually result in immunosuppression by exhausting immune cells or disrupting immune regulatory pathways (Kanterman et al., 2012). This suggests that exposure to FTN pyrethroid affects the specific pro-inflammatory cytokines that mediate the immune system’s intercellular communication.

Immunoglobulins (IgG and IgM) were examined in this study as indicators of the specific immunological response (Abd-Elhakim et al., 2018b; Abd-Elhakim et al., 2020b). Activated B-cells secrete them and serve several immunological functions (Abd-Elhakim et al., 2016; Abd-Elhakim et al., 2018c). IgM and IgG are primarily involved in the clearance of antigens (Khaled Abo-El-Soouda et al., 2018; Abo-El-Sooud et al., 2019). Thus, the observed increase in IgM levels in CML-CNP group may be attributed to CML-CNP’s ability to activate B lymphocytes, enhancing the production of primary immunoglobulin (IgM) as part of the humoral immune response (Mohammadi et al., 2022). This effect is likely mediated by the nanoparticles’ role in mitigating oxidative stress and inflammation in the splenic microenvironment, as evidenced by the increase in the antioxidant (GSH) and the reduction tendency in the pro-inflammatory cytokines (IL-1β). Additionally, the improved splenic histology and upregulation of CD20 (a B-cell marker) suggest a supportive role of CML-CNP in B-cell activation and function (Pavlasova and Mraz, 2020). On the contrary, the FTN-exposed rats significantly reduced serum levels of IgG, not IgM. Similarly, high doses of cypermethrin and deltamethrin induced immunosuppressive effects on cell-mediated and humoral immune responses in rats (Tulinská et al., 1995) and mice (Haratym-Maj et al., 2006). Immunoglobulin concentration declines indicate that the specific immune system may be impacted and are mainly related to lymphocyte populations (Motwadie et al., 2021; Hashem et al., 2022). In our study, the FTN-exposed rats exhibited an obvious lymphocytopenia accompanied by a substantial downregulation of CD4, CD3, CD20, and CD56 but upregulation of CD8. Comparably, pyrethroid treatment of activated human cells *in vitro* or *ex vivo* inhibited lymphocyte proliferation, depending on concentration (Diel et al., 1999). T-cell counts and subpopulations could be used to assess cell-mediated immunity (Actor, 2023). They are distinguished by surface indicators known as clusters of differentiation (CD). This surface marker is known to identify mature lymphocytes (CD3) (Pavelek et al., 2020), T-helper/inducer cells (CD4), and T-suppressor/cytotoxic cells (CD8) (Hashem et al., 2020; Manusama et al., 2023). Analysing B-cells (CD20) is important in evaluating antibody-mediated immunity (Pavlasova and Mraz, 2020). CD56 cells are natural killer cells, which play a crucial role in non-specific immunity by eliminating diseased cells (Gianchecchi et al., 2018). Of note, the downregulation of CD cells was accompanied by the upregulation of the pro-inflammatory cytokines genes (TNF-α and IL-6). In this respect, a growing body of data declared that pro-inflammatory mediators are the primary drivers of immune dysfunction and how this understanding could help shed light on the seeming contradiction of immune suppression in a patient exhibiting hyperinflammation symptoms (Morris et al., 2013; Liu et al., 2020). Additionally, it was shown by Shimizu et al. (2018) that macrophages primarily release a panel of inflammatory cytokines, including IL6, IL1β, and transforming growth factor, which induce immunological suppression. Another contributing factor that could participate in the FTN-induced immunosuppression could be the previously reported hyperlipidemia (Alqahtani et al., 2023) and increased corticosterone associated with pyrethroid exposure (Righi et al., 2009). Similarly, reduced IgG, but not IgM, has been reported to be related to dyslipidemia (Lin et al., 2019). In this respect, Lin et al. (2019) demonstrated that IgG glycosylation patterns are markedly correlated with blood lipid levels, implying that alterations in IgG structure may signal underlying dyslipidemia. Moreover, autoantibodies like anti-ApoA-1 IgG have been demonstrated to affect cholesterol homeostasis and foam cell production (Pagano et al., 2019). Furthermore, Liu et al. (2018) reported that the depletion of sialic acid and galactose in IgG correlates with chronic inflammation in dyslipidemia. Yet, further mechanistic studies should focus on elucidating the other potential pathways responsible for FTN-induced immune dysfunction, particularly the stability of IgM levels despite reductions in IgG and lymphocyte populations.

In the current experiment, CML-CNP oral dosing significantly counteracted FTN-induced immunosuppression and inflammatory reactions, as evidenced by the hematological, biochemical, immunohistochemical, and molecular analysis. Similarly, CML-CNP considerably prohibited macrophage-induced inflammatory responses *in vitro* through deactivating NF-κB and down-regulating pro-inflammatory cytokines (IL-6 and TNF-α) (Li et al., 2019). Moreover, CMN has an anti-inflammatory effect through decreasing TNF-α (Yon et al., 2016). Several reasons could be behind the immune-stimulant and anti-inflammatory activity of CML-CNP. Initially, the antioxidant activity of CML-CNP (evidenced by decreased ROS and increased GSH and GPX levels) could be responsible for the restoration of splenic architecture (evinced histopathologically), with the critical role of the spleen in immunity (Lewis et al., 2019), and consequently immunostimulant activity. Also, as the liver is responsible for producing most of the circulating innate immunity proteins in the body thus, CML-CNP hepatoprotective activity (Alqahtani et al., 2023) could be another possible cause. In addition, CMN can lower the incidence of inflammatory reactions by scavenging ROS, inhibiting lipid peroxidation (Panahi et al., 2018).

The FTN-exposed rats showed an evident hypochromic normocytic anemia reflected in reduced RBC count, Hb, and MCV, with no significant change in MCV but increased MCH and MCHC. This could be related to the altered metabolism of folate (Curtis et al., 2023) or hyperthyroidism (Kaul et al., 1996; Chang et al., 2018) associated with pyrethroid exposure. Moreover, the FTN-induced hepatotoxic effect could be a critical predisposing factor for this type of anemia (Alqahtani et al., 2023). Corroborating these findings, the histopathological findings revealed a marked hemosiderosis in the spleen of the FTN-exposed group. Hemosiderosis refers to the excess iron deposition in the spleen, which could result from red cell destruction or the production of abnormal fragile RBCS. In this line, several pyrethroids have been reported to cause oxidative damage to RBCs or produce RBCs of aberrant morphologies (Khan et al., 2012). On the other hand, a significant correction of the erythrogram components and reduced splenic hemosiderosis was obvious in the FTN + CML-CNP-treated rats. In this respect, in the recent *in vitro* study of Asif et al. (2023), CML-CNP showed a dose-dependent increase in percentage protection of human RBC membrane stabilization and anti-inflammatory activity. Previous research found that substances with membrane-stabilizing qualities can interfere with phospholipase release, which causes the generation of inflammatory mediators. Numerous plants with anti-inflammatory properties can also reduce thermally induced protein denaturation (Rahman et al., 2015). Additionally, the hepatoprotective activity of CML-CNP (Alqahtani et al., 2023) could partly play a role in counteracting FTN-induced anemia. Moreover, restoring splenic architecture with its roles in red blood cell clearance and hematopoiesis (Lewis et al., 2019) could contribute to balancing FTN-inducing anemia.

Of note, FTN exposure induced an obvious thrombocytosis in the current experiment. Several factors could contribute directly to FTN-induced more significant platelet activity, including metabolic abnormalities such as hyperglycemia (Kim et al., 2015) and hyperlipidemia (Alqahtani et al., 2023), as well as associated conditions such as oxidative stress, inflammation, and endothelial dysfunction (Jaremek and Nieradko-Iwanicka, 2020). Additionally, the histopathological findings revealed endothelial hypertrophy and increased numbers of megakaryocytes, the precursor cells for platelets, in the FTN-exposed group. Interestingly, despite the previously documented favorable effects of CML-CNP on blood glucose balance (Sudirman et al., 2019), endothelial cell function (Sudirman et al., 2019), and lipid profile (Alqahtani et al., 2023), thrombocytosis was still found in the FTN + CML-CNP-treated rats. Besides, the increment in megakaryocytes was also detected histopathologically in the FTN + CML-CNP-treated rats. The possible explanation for this finding is that this is a favorable defense of thrombocytosis associated with improved splenic architecture and reduced hemosiderosis, unlike that detected in the FTN group. In line with this, platelets have been reported to participate in innate immunity due to their ability to release many bioactive molecules stored within granules or synthesized upon activation. These mediators recruit and modulate the innate immune system’s effector cells. Furthermore, platelets have a direct effector function and are considered effector cells in innate immunity (Ali et al., 2015).

Herein, splenic oxidative stress, lipid peroxidation, and apoptotic reactions were induced by FTN. This was demonstrated by a significant decrease in the antioxidant indices (GSH, GPX, and GSH/GSSG) and an increment of the lipid peroxidation marker (MDA) and the apoptotic protein (Caspase-3) immunoexpression in the splenic tissues. Similarly, FTN-induced oxidative stress and apoptotic reactions were recorded in different organs, including the testis (Mohamed et al., 2019) and liver (Alqahtani et al., 2023). Comparably, in an *in vitro* experiment using murine macrophages, Cis-bifenthrin reduced cell viability and prompted apoptosis through downregulating Bcl-2 and upregulating caspase 3 and p53 (Wang et al., 2017). Likewise, studies on thymocytes and splenocytes from mice have demonstrated that deltamethrin causes oxidative stress and apoptosis (Kumar et al., 2014; Kumar and Sharma, 2015). In this regard, some type II pyrethroids, such as deltamethrin, cypermethrin, and lambda-cyhalothrin, have oxidative stress as their primary harmful mechanism (Chargui et al., 2010; Das et al., 2016; Deeba et al., 2017). Type II pyrethroids are characterized by a secondary alcohol ester that has a cyano group at the α-carbon of the alcohol moiety (Anadon et al., 2006). Consequently, their ability to induce oxidative stress may be linked to the formation of chemically unstable cyanohydrins. These compounds were subsequently transformed into aldehydes and cyanides, which, in turn, supplied free radicals. Since cyanohydrins decompose into free radical-producing cyanides and aldehydes, their capacity to induce oxidative stress may stem from this process (Abu Zeid et al., 2021). The lipophilicity and cell membrane-crossing capabilities of type II pyrethroids also raise the possibility that they cause lipid peroxidation (Prasanthi and Rajini, 2005). ROS production during pyrethroid metabolism can potentially trigger lipid peroxidation (Abu Zeid et al., 2021). Nevertheless, CML-CNP treatment significantly reduced lipid peroxidation, oxidative stress, and apoptotic events in FTN-intoxicated rats. This was achieved by increasing their GPX and GSH contents while simultaneously decreasing MDA levels and Caspase-3 immunoexpression in their splenic tissues. The antioxidant capabilities of CM and its nano form may be due, in part, to their ability to inhibit ROS-producing enzymes such as microsomal monooxygenase, mitochondrial succinoxidase, and NADH oxidase (Alisi et al., 2018). In addition, CM might increase the antioxidant enzymes activity and scavenge free radicals due to its conjugated structure and enol form (Trujillo et al., 2013). In the presence of toxicants, CM also helps to prevent peroxidation, which harms the cell membrane (Sankar et al., 2012). These findings led to the hypothesis that CM protected organs by halting lipid peroxidation and maintaining the integrity of cell membranes (Mathew et al., 2012). This is accomplished by controlling transcription factors, inflammatory cytokines, and signal transduction pathways.

A key point to consider is that the current investigation used a commercial FTN product to imitate practical exposure. This commercial product comprises 80% unspecified substances, the identities of which are kept by the manufacturer as trade secrets. Emulsifying agents and other additives are commonly included in emulsifiable formulations (Cush, 2006). Previous studies indicated that numerous commercial pesticide formulations contain petroleum distillates or hydrocarbons as solvent carriers (Jungers et al., 2022). Reports have connected recurrent and sustained occupational solvent exposure to brain and nervous system injury (Dick, 2006; Eddleston et al., 2012). However, the reports are unclear on the exact solvents that could induce these symptoms. Accordingly, the potential implications of the solvent used in the production of FTN commercial items in the recorded adverse neurobehavioral consequences warrant further research.

## 5 Conclusion

The study findings provide insights into the interaction between inflammatory cytokines and immune components in the context of FTN-induced inflammation-immunosuppression, highlighting the need for further research to fully understand the mechanisms and associated risks. Moreover, this study is the first one demonstrating the favorable immune-modulatory role of CML-CNP against FTN-induced hemato-imunnological disturbances, probably via the antioxidant, anti-apoptotic, and anti-inflammatory mechanisms. This approach highlights the potential of chitosan as a component of drug delivery systems for regulated and prolonged CMN release, although further studies are needed to assess its pharmacokinetics and bioavailability *in vivo.* Thus, the findings provide credence to the need for further evaluations of FTN risks through dose-response studies and the identification of therapeutic targets for CML-CNP clinical treatment. Additionally, human studies are needed to validate these findings in clinical settings, accounting for species-specific differences in immune responses to find actionable solutions for public health.

## Data Availability

The original contributions presented in the study are included in the article/supplementary material, further inquiries can be directed to the corresponding author.
